# SARS-CoV-2 infection and COVID-19 vaccination rates in pregnant women in Scotland

**DOI:** 10.1038/s41591-021-01666-2

**Published:** 2022-01-13

**Authors:** Sarah J. Stock, Jade Carruthers, Clara Calvert, Cheryl Denny, Jack Donaghy, Anna Goulding, Lisa E. M. Hopcroft, Leanne Hopkins, Terry McLaughlin, Jiafeng Pan, Ting Shi, Bob Taylor, Utkarsh Agrawal, Bonnie Auyeung, Srinivasa Vittal Katikireddi, Colin McCowan, Josie Murray, Colin R. Simpson, Chris Robertson, Eleftheria Vasileiou, Aziz Sheikh, Rachael Wood

**Affiliations:** 1 University of Edinburgh Usher Institute, Edinburgh, UK; 2 Public Health Scotland, Scotland, UK; 3 The DataLab, Nuffield Department of Primary Care Health Sciences, University of Oxford, Oxford, UK; 4 School of Medicine, University of St Andrews, St Andrews, UK; 5 School of Philosophy, Psychology and Language Sciences, University of Edinburgh, Edinburgh, UK; 6 MRC/CSO Social & Public Health Sciences Unit, University of Glasgow, Glasgow, UK; 7 Institute of Health & Wellbeing, University of Glasgow, Glasgow, UK; 8 School of Health, Wellington Faculty of Health, Victoria University of Wellington, Wellington, New Zealand; 9 Department of Mathematics and Statistics, University of Strathclyde, Glasgow, UK

**Keywords:** Viral infection, Epidemiology, Epidemiology

## Abstract

Population-level data on COVID-19 vaccine uptake in pregnancy and SARS-CoV-2 infection outcomes are lacking. We describe COVID-19 vaccine uptake and SARS-CoV-2 infection in pregnant women in Scotland, using whole-population data from a national, prospective cohort. Between the start of a COVID-19 vaccine program in Scotland, on 8 December 2020 and 31 October 2021, 25,917 COVID-19 vaccinations were given to 18,457 pregnant women. Vaccine coverage was substantially lower in pregnant women than in the general female population of 18−44 years; 32.3% of women giving birth in October 2021 had two doses of vaccine compared to 77.4% in all women. The extended perinatal mortality rate for women who gave birth within 28 d of a COVID-19 diagnosis was 22.6 per 1,000 births (95% CI 12.9−38.5; pandemic background rate 5.6 per 1,000 births; 452 out of 80,456; 95% CI 5.1−6.2). Overall, 77.4% (3,833 out of 4,950; 95% CI 76.2−78.6) of SARS-CoV-2 infections, 90.9% (748 out of 823; 95% CI 88.7−92.7) of SARS-CoV-2 associated with hospital admission and 98% (102 out of 104; 95% CI 92.5−99.7) of SARS-CoV-2 associated with critical care admission, as well as all baby deaths, occurred in pregnant women who were unvaccinated at the time of COVID-19 diagnosis. Addressing low vaccine uptake rates in pregnant women is imperative to protect the health of women and babies in the ongoing pandemic.

## Abstract (web summary)

Findings from the COVID-19 in Pregnancy in Scotland (COPS) study reveals low levels of vaccination uptake by pregnant women compared to women in the general population and that not being vaccinated is associated with increased risk of severe complications of COVID-19 in pregnancy, including perinatal mortality.

## Main

Comprehensive whole-population data on COVID-19 vaccine uptake in pregnancy and SARS-CoV-2 infection rates and COVID-19 outcomes are lacking^1^. Such data are required to help guide decision making by women, clinicians and policymakers on measures to prevent and control COVID-19 in pregnancy, in particular, vaccination.

The first case of SARS-CoV-2 in Scotland was identified on 1 March 2020. Different SARS-CoV-2 variants have predominated in subsequent waves of infection, with wild type predominant initially, the Alpha variant dominant from January 2021 and the Delta variant dominant from May 2021. SARS-CoV-2 testing was initially restricted due to limited availability, with availability in the community for any symptomatic adult available from 18 May 2020. Although not uniformly implemented in all maternity units, routine SARS-CoV-2 testing of maternity admissions was instituted from 1 December 2020.

Pregnant women do not seem to be more susceptible to SARS-CoV-2 infection than non-pregnant women, but they are at higher risk of severe COVID-19 disease^2–4^. Compared to non-pregnant women of reproductive age, pregnant women with SARS-CoV-2 infection are more likely to be admitted to critical care, receive invasive ventilation and extracorporeal membrane oxygenation and die^2,3^. COVID-19 in pregnancy is associated with increased risk of the pregnancy specific complications pre-eclampsia, preterm birth and stillbirth^2,5–10^.

Despite widespread recognition of potential vulnerability to COVID-19, pregnant women were excluded from pre-marketing clinical trials studying COVID-19 vaccines^11,12^. As a result, evidence to inform decision making on vaccination was largely absent when vaccination programs started and recommendations on vaccination in pregnancy have varied over time as well as by country^13^. Post-marketing surveillance data suggest that COVID-19 vaccine effectiveness is broadly similar to that in non-pregnant individuals^14,15^. Data on the safety of vaccines in pregnancy comes from preclinical studies of COVID-19 vaccines in animals^16^, findings from women who had unanticipated pregnancies while participating in clinical trials^17^ and accumulating pharmacovigilance data^18–21^, all of which are reassuring regarding COVID-19 vaccine safety in pregnancy.

**Fig. 1:**
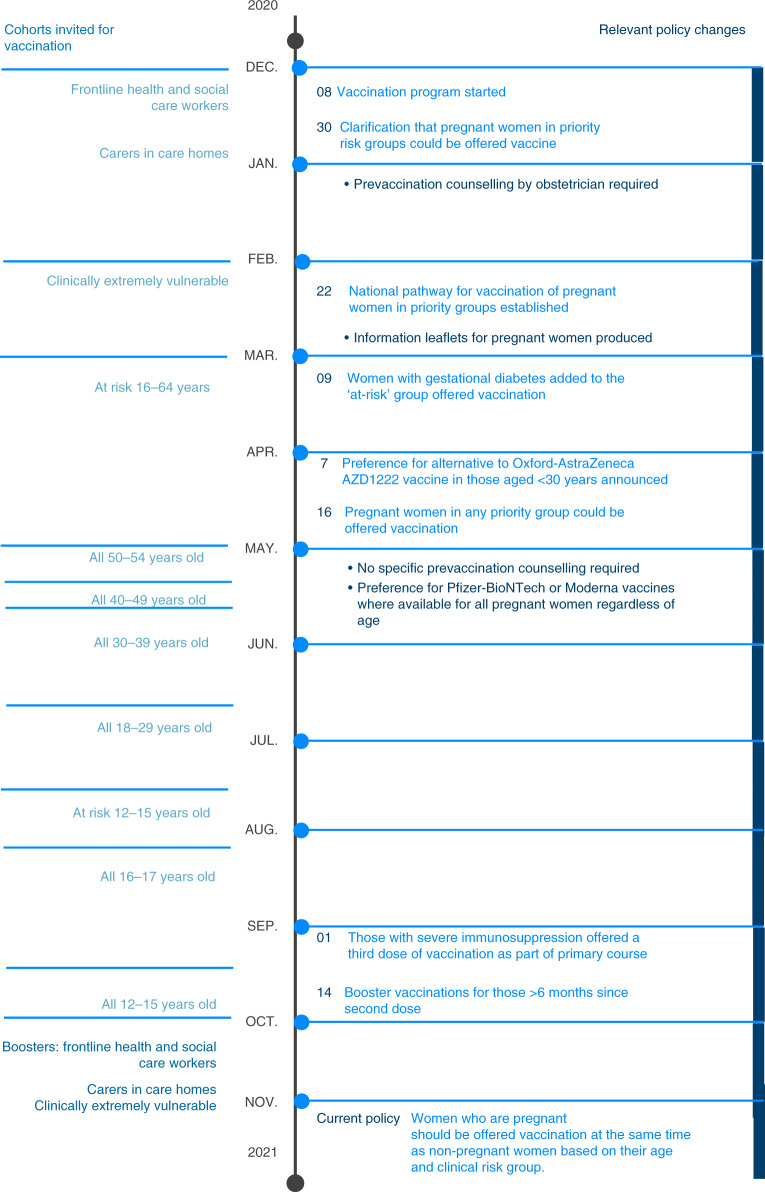
Populations invited for COVID-19 vaccination in Scotland over time. Summary of population cohorts invited for COVID-19 vaccination over time in Scotland from the start of the vaccination program on 8 December 2020 (left) and policy changes relevant to vaccination in pregnant women (right).

The COVID-19 vaccination program in Scotland commenced on 8 December 2020 and policies on the provision of COVID-19 vaccination to pregnant women evolved over time (Fig. 1). From 16 April 2021 the recommendation has been that women who are pregnant should be offered vaccination at the same time as non-pregnant women, based on their age and clinical risk group and no pregnancy specific pre-vaccination counseling is required.

The COVID-19 in Pregnancy in Scotland (COPS) study (a sub-study of EAVE II (Early Pandemic Evaluation and Enhanced Surveillance of COVID-19)^22,23^) is a national, prospective dynamic cohort of all women who were pregnant on, or became pregnant after, 1 March 2020; linked to SARS-CoV-2 infection data and COVID-19 vaccination data^24,25^. The aims of this study were to use data from the COPS cohort to describe the uptake and coverage of COVID-19 vaccination in pregnant women; to describe the incidence of SARS-CoV-2 infection in pregnant women; to describe rates of hospital admission, critical care admission, preterm birth and extended perinatal mortality following SARS-CoV-2 infection in pregnancy; and to explore the effect of COVID-19 vaccination status on these post-infection outcomes.

## Results

### Cohort

**Fig. 2:**
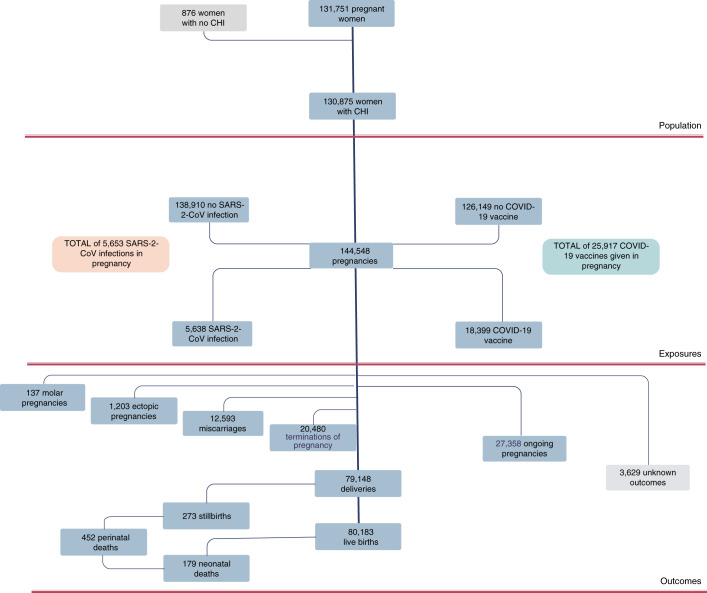
Summary of participants in the COVID-19 in Pregnancy in Scotland cohort. Flow chart summarizing participants in the COPS cohort. Counts for molar pregnancies, ectopic pregnancies, miscarriages, terminations and deliveries are numbers of pregnancies. Live births, stillbirths and neonatal deaths are numbers of babies.

We used the COPS database linked to records of COVID-19 vaccinations delivered and SARS-CoV-2 infections diagnosed up to and including 31 October 2021. This included data on a total of 145,424 pregnancies in 131,751 women. Overall, 144,548 (99.4%) of these pregnancy records (and 130,875, 99.3% of the women) had an associated unique community health index (CHI) number used for data linkage. Pregnancy records with no associated CHI number are likely to relate to duplicate records so were excluded from this analysis. Figure 2 describes the participants in the cohort. Overall, 117,190 pregnancies were complete, with 13,933 ending in early pregnancy loss (miscarriage, molar or ectopic pregnancy), 20,480 resulting in termination of pregnancy, 79,148 resulting in delivery and 3,629 having an unknown outcome. The deliveries resulted in 273 stillbirths and 80,183 live births, of which 179 resulted in neonatal death. Overall, 27,358 pregnancies were ongoing on 31 October 2021.

Pregnancy data were linked with national data on confirmed SARS-CoV-2 infection and COVID-19 vaccination using CHI number. Overall, 99% of all the PCR with reverse transcription (RT−PCR) samples within the national testing database since the start of the pandemic, and 99.8% of all records for vaccinations given from the start of the program, have an associated CHI number.

Infection and vaccination in pregnancy was defined as infection diagnosed or vaccination given at any point from the date of conception (2 + 0 weeks gestation) to the date the pregnancy ends inclusive. The date of first positive viral RT−PCR sample collection was taken as the date of onset of the first episode of COVID-19. Subsequent episodes were recorded if a positive viral RT−PCR sample was taken ≥90 d after a first positive sample.

To explore the impact of vaccination status on SARS-CoV-2 infection, women were grouped as follows: unvaccinated (no previous COVID-19 vaccination before the date of onset of COVID-19 or with one dose of vaccination ≤21 d before the date of onset); partially vaccinated (one dose of vaccination >21 d before the date of onset of COVID-19 or two doses of vaccination with the second dose administered ≤14 d before the date of onset); or fully vaccinated (two doses of vaccination with the second dose >14 d before the date of onset of COVID-19). We also report the number of third or booster doses given, but have not included these in definitions of vaccination status, as third and booster doses in Scotland were only recommended from 14 and 20 September 2021, respectively.

### COVID-19 vaccine coverage and uptake in pregnancy

From 1 December 2020 to 31 October 2021 a total of 25,917 COVID-19 vaccinations (12,518 (48%) first doses, 12,194 (47%) second doses and 1,205 (5%) third or booster doses) had been given in 18,399 pregnancies. A total of 9,905 (38.2%; 95% CI 37.6−38.8) vaccinations were given in the first trimester of pregnancy (at 2 + 0 to 13 + 6 weeks gestation); 9,317 (35.9%; 95% CI 35.4−36.5) in the second trimester (at 14 + 0 to 27 + 6 weeks gestation); and 6,695 (25.8%; 95% CI 25.3−26.4) in the third trimester (at 28 + 0 weeks gestation or over). Of the vaccinations given, 20,572 (79.4%; 95% CI 78.9−79.9) were Pfizer-BioNTech BNT162b2 messenger RNA vaccine; 3,224 (12.4%; 95% CI 12.0−12.8) were Moderna mRNA-1273 mRNA vaccine; and 2,121 (8.2%; 95% CI 7.9−8.5) were Oxford-AstraZeneca AZD1222 viral vector vaccine (given mainly early in the vaccine program to pregnant women with a clinical risk factor indicating vaccination).

We used two different metrics to describe vaccination in pregnancy, monthly uptake in women who were pregnant during the month and coverage (the proportion of women who, at the time of giving birth, had been vaccinated, whether vaccination was before or during pregnancy). Uptake and coverage data in the general female population of reproductive age (18−44 years) were provided for context, generated from data collected by Public Health Scotland^26^.

**Fig. 3:**
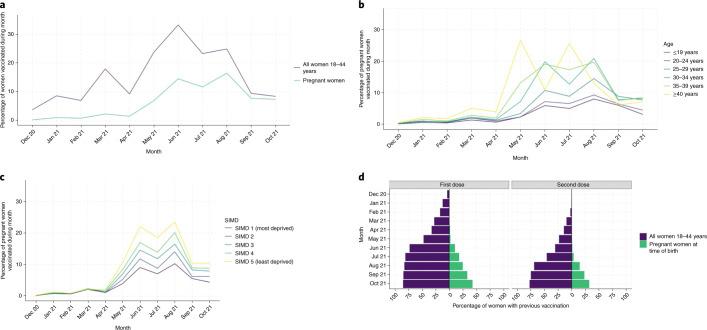
COVID-19 vaccination uptake and coverage. **a**, Monthly uptake of COVID-19 vaccination in all women 18−44 years and in pregnant women. **b**, Monthly uptake of COVID-19 vaccination in pregnant women by age group. **c**, Monthly uptake of COVID-19 vaccination in pregnant women by SIMD quintile. SIMD, Scottish Index Multiple Deprivation, with SIMD 5 being least deprived and 1 being most deprived. **d**, Vaccine coverage in the general female population in women age 18−44 years (purple) and percentage of pregnant women vaccinated before the time of birth (green). Numbers of women 18−44 years were derived from 2020 mid-year population estimates^26^.

COVID-19 vaccine uptake among pregnant women each month was consistently lower than that in the general female population of reproductive age (Fig. 3a). Vaccine uptake was consistently lowest in younger (≤20 years) pregnant women and those living in the most deprived areas of Scotland (Fig. 3b,c).

COVID-19 vaccine coverage increased over time, but has consistently been substantially lower among pregnant women than in the general female population of reproductive age (Fig. 3d). In October 2021, 4,064 women gave birth, of whom 1,738 (42.8%; 95% CI 41.2−44.3) had received any COVID-19 vaccination before delivery, with 1,311 (32.3%; 95% CI 30.8−33.7) of the women having received two primary doses of vaccination and 36 women (0.9%; 95% CI 0.6−1.2) having received a third or booster dose of vaccination. In contrast, by 31 October 2021, 84.7% (803,241 out of 947,984; 95% CI 84.7−84.8) of women aged 18–44 years in the general population had received any vaccination, 77.4% had received two doses (733,942 out of 947,984; 95% CI 77.3−77.5) and 7.0% had received a third dose or booster dose (66,001 out of 947,984; 95% CI 6.9−7.0).

### SARS-CoV-2 outcomes in vaccinated and unvaccinated pregnant women

**Fig. 4:**
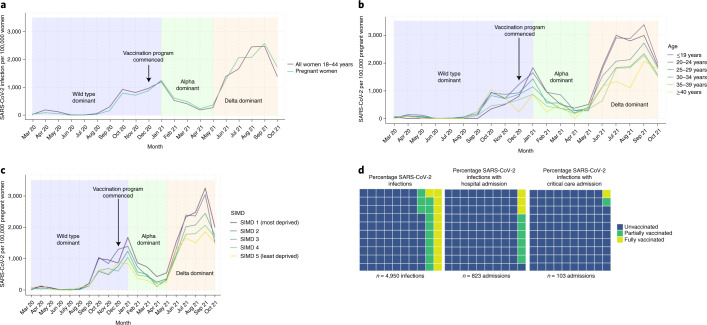
SARS-CoV-2 in pregnancy and outcomes by vaccination status. **a**, SARS-CoV-2 in all women 18−44 years per 100,000 women and in pregnant women. **b**, Monthly rates of SARS-CoV-2 per 100,000 pregnant women by age group **c**, Monthly rates of SARS-CoV-2 per 100,000 pregnant women by SIMD. **d**, Percentage of cases of SARS-CoV-2 infection in pregnancy occurring 1 December 2020 to 31 October 2021 inclusive, cases with associated hospital admission and cases with associated critical care admission, by vaccination status at the date of onset of COVID-19. Unvaccinated is defined as no COVID-19 vaccination before the date of onset of COVID-19 or one dose of vaccination ≤21 d previously in line with standard definitions used by Public Health Scotland.

Between 1 March 2020 and 31 October 2021, there were 5,653 confirmed SARS-CoV-2 infections in pregnancy. Overall, rates of SARS-CoV-2 infection in pregnancy showed similar patterns to those in the general female population of reproductive age, with peaks of infection in October 2020, January 2021 and September 2021 (Fig. 4a). SARS-CoV-2 infection rates have been consistently highest in pregnant women living in the most deprived areas and in younger, compared to older, pregnant women (Fig. 4b,c).

Early in the pandemic, it is highly likely that there was under-ascertainment of cases of SARS-CoV-2 infection due to limited test availability and testing capacity (Fig. 4a). Restricting the COPS cohort to only data from 1 December 2020 onward (the date that routine SARS-CoV-2 testing was recommended for all admissions to maternity care and the COVID-19 vaccination program started in Scotland) reduces the size of the cohort, but allows more comparable estimation of rates of severe outcomes (hospital admission, critical care admission and perinatal mortality) associated with SARS-CoV-2 infection in vaccinated and unvaccinated women. From 1 December 2020 onward, the COPS database included linked data on a total of 91,183 pregnancies in 87,694 women.

There were 4,950 confirmed SARS-CoV-2 infections in pregnancy from 1 December 2020. SARS-CoV-2 infections were relatively evenly spread throughout pregnancy (1,543 (31.2%; 95% CI 29.9−32.5) diagnosed in the first trimester of pregnancy; 1,850 (37.4%; 95% CI 36.0−38.7) in the second trimester; and 1,557 (31.5%; 95% CI 30.2−32.8) in the third trimester).

Overall, 823 of the 4,950 SARS-CoV-2 infections in pregnancy from 1 December 2020 onward (16.6%; 95% CI 15.6−17.7) were associated with any hospital admission (date of onset of infection occurred during a hospital admission or within 14 d before admission) and 104 SARS-CoV-2 infections in pregnancy (2.1%; 95% CI 1.7−2.6) were associated with a critical care admission (date of onset of infection occurred during a critical care admission or within 21 d before admission). In first trimester SARS-CoV-2 infections, 6.7% (103 out of 1,543; 95% CI 5.5−8.1) were associated with any hospital admission, compared to 10.7% (198 out of 1,850 cases; 95% CI 9.3−12.2) of those in the second trimester and 33.5% (522 out of 1,557; 95% CI 31.2−35.9) of those in the third trimester. No (0 out of 1,543 cases; 95% CI 0−0.3) SARS-CoV-2 infections in the first trimester were associated with critical care admission, compared to 2.0% (37 out of 1,850 cases; 95% CI 1.4−2.8) of those in the second trimester and 4.3% (67 out of 1,557; 95% CI 3.4−5.5) of those in the third trimester.

From 1 December 2020 onward, 77.4% of SARS-CoV-2 infections in pregnancy (3,833 out of 4,950; 95% CI 76.2−78.6) occurred in women who were unvaccinated at the date of onset of infection, with 11.5% (567 out of 4,950; 95% CI 10.6−12.4) in partially vaccinated women and 11.1% (550 out of 4,950; 95% CI 10.3−12.0) in fully vaccinated women.

Of the SARS-CoV-2 infections in pregnancy that occurred in unvaccinated women, 19.5% (748 out of 3,833; 95% CI 18.3−20.8) were associated with hospital admission, compared to 8.3% (47 out of 567; 95% CI 6.2−10.9) of those in partially vaccinated women and 5.1% (28 out of 550; 95% CI 3.5−7.4) of those in fully vaccinated women. A total of 2.7% (102 out of 3,833; 95% CI 2.2−3.2) of the SARS-CoV-2 infections in pregnancy that occurred in unvaccinated women were associated with a critical care admission, compared to 0.2% (1 out of 567; 95% CI 0.01−1.1) of those in partially vaccinated women and 0.2% (1 out of 550 cases; 95% CI 0.01−1.2) of those in fully vaccinated women. This means that while 77.4% (3,833 out of 4,950; 95% CI 76.2−78.6) of SARS-CoV-2 infections in pregnancy occurred in unvaccinated women, 90.9% (748 out of 823; 95% CI 88.7−92.7) of infections associated with hospital admission and 98.1% (102 out of 104; 95% CI 92.5−99.7) of infections associated with critical care admission, were in unvaccinated women (Fig. 4d). To date, there has been one maternal death following SARS-CoV-2 infection in pregnancy in Scotland.

A total of 2,364 babies have been born to women who had SARS-CoV-2 infection in pregnancy between 1 December 2020 and 31 October 2021. Of these, 2,353 were live births, of which 241 were preterm births (<37 weeks gestation; preterm birth rate 10.2%; 95% CI 9.1−11.6). Overall, 610 of the live births and 101 of the preterm births occurred within 28 d of the date of onset of the mother’s SARS-CoV-2 infection, giving a preterm birth rate among babies born within 28 d of SARS-CoV-2 infection of 16.6% (95% CI 13.7−19.8).

Of the 2,364 total births, 11 were stillbirths (deaths in utero ≥ 24 weeks gestation) and eight live births resulted in neonatal deaths (death within 28 d of birth), giving an extended perinatal mortality rate of 8.0 per 1,000 births following SARS-CoV-2 infection at any point in pregnancy (19 out of 2,364; 95% CI 5.0−12.8). Ten of the stillbirths and four neonatal deaths occurred in babies born within 28 d of the onset of maternal infection, giving an extended perinatal mortality rate of 22.6 per 1,000 births (14 out of 620, 95% 12.9−38.5) in this population. All perinatal deaths following SARS-CoV-2 infection in pregnancy occurred in women who were unvaccinated at the time of SARS-CoV-2 infection. We do not have access to detailed clinical records to assess whether COVID-19 directly or indirectly contributed to the preterm births and deaths seen following maternal infection.

**Fig. 5:**
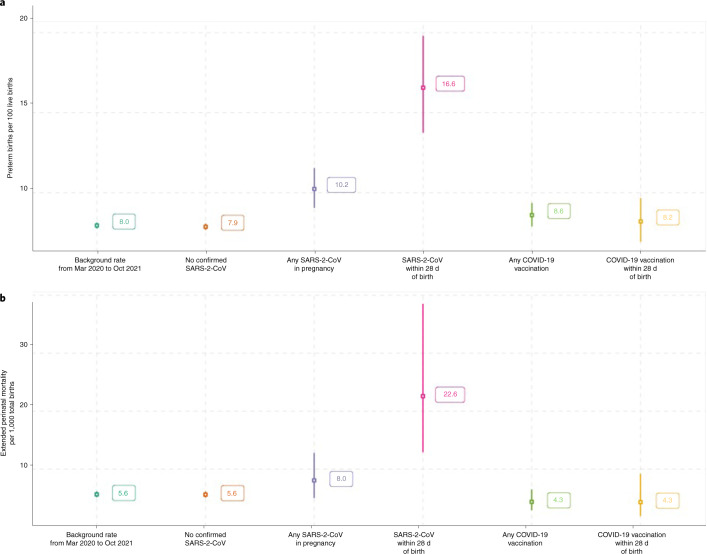
Preterm birth and perinatal mortality after SARS-CoV-2 in pregnancy. **a**,**b**, Preterm birth (<37 weeks gestation) rate per 100 live births (**a**) and extended perinatal mortality rates (**b**) (stillbirths (death in utero ≥24 weeks gestation) and neonatal deaths within 28 d of birth per 1,000 total births) in different cohorts during the pandemic. Background rate is the rate for all babies born during the pandemic period (1 March 2020 to 31 October 2021); no confirmed SARS-CoV-2 is the rate for babies born during the pandemic period (1 March 2020 to 31 October 2021) to women with no confirmed SARS-CoV-2 infection during pregnancy; any SARS-CoV-2 is the rate for babies born to women who had confirmed SARS-CoV-2 during their pregnancy 1 December 2020 to 31 October 2021; SARS-CoV-2 within 28 d of birth is the rate for babies born to women who had confirmed SARS-CoV-2 during their pregnancy 1 December 2020 to 31 October 2021, restricted to babies born within 28 d of the date of onset of maternal infection; Any COVID-19 vaccination is the rate for babies born to women who had COVID-19 vaccination during their pregnancy 1 December 2020 to 31 October 2021; COVID-19 vaccination within 28 d of birth is the rate for babies born to women who had COVID-19 vaccination during their pregnancy 1 December 2020 to 31 October 2021, restricted to babies born within 28 d of the date of maternal vaccination.

For comparison, the background preterm birth rate during the pandemic (from 1 March 2020 to 31 October 2021) was 8.0% (6,381 out of 80,183 live births; 95% CI 7.8−8.1) and the extended perinatal mortality rate was 5.6 per 1,000 births (452 out of 80,456 total births; 95% CI 5.1−6.2). When restricted to babies born to women with no confirmed SARS-CoV-2 infection during pregnancy the preterm birth rate was 7.9% (6,083 out of 77,209 live births; 95% CI 7.7−8.1) and the extended perinatal mortality was 5.6 per 1,000 births (432 out of 77,470 total births; 95% CI 5.1−6.1). These preterm and perinatal mortality rates are shown in Fig. 5, along with the preterm birth and extended perinatal mortality rates in women who received the COVID-19 vaccine in pregnancy (preterm birth rate of 8.6% (495 out of 5,752 live births in women who received COVID-19 vaccination in pregnancy; 95% CI 7.9−9.4) and 8.2% (134 out of 1,632 live births within 28 d of COVID-19 vaccination in pregnancy; 95% CI 7.0−9.6); and extended perinatal mortality of 4.3 per 1,000 births (25 out of 5,766 total births in women who received COVID-19 vaccination in pregnancy; 95% CI 2.9−6.4) and 4.3 per 1,000 births (7 out of 1,635 births within 28 d of COVID-19 vaccination; 95% CI 1.9−9.2)).

## Discussion

These population data provide new national evidence of low levels of vaccine uptake in pregnancy in Scotland, at a time when the Delta variant dominated, with only 32.3% of pregnant women giving birth in October having had two doses of the vaccine. Rates were lowest in younger women and populations who lived in the most deprived areas. SARS-CoV-2 infection rates in pregnancy closely mirrored those in the general population throughout the pandemic, when different SARS-CoV-2 variants predominated in Scotland (wild type initially, Alpha dominant from January 2021 and Delta dominant from May 2021). We found that severe complications known to be associated with COVID-19 in pregnancy (critical care admission and perinatal mortality) were more common in women who were unvaccinated at the time of COVID-19 diagnosis than in vaccinated pregnant women. Our data support the importance of women being vaccinated in pregnancy to prevent adverse outcomes associated with COVID-19. For extended perinatal mortality, for example, we find substantially elevated rates among women who had SARS-CoV-2 within 28 d of birth; conversely, for women who had COVID-19 vaccination, extended perinatal morality rates were similar to background rates and among women with no confirmed SARS-CoV-2.

Although surveillance studies, which have mainly been based on women admitted to hospital, have suggested that pregnant women are at highest risk of severe COVID-19 disease in the third trimester^9^, these Scottish population data, capturing all virologically confirmed infections in a setting where testing has been widely available in hospital and the community, show that SARS-CoV-2 infections were relatively evenly spread throughout pregnancy. However, in line with findings from other studies, SARS-CoV-2 associated with hospital and critical care admission was more common as pregnancy progressed^4,9^.

**Fig. 6:**
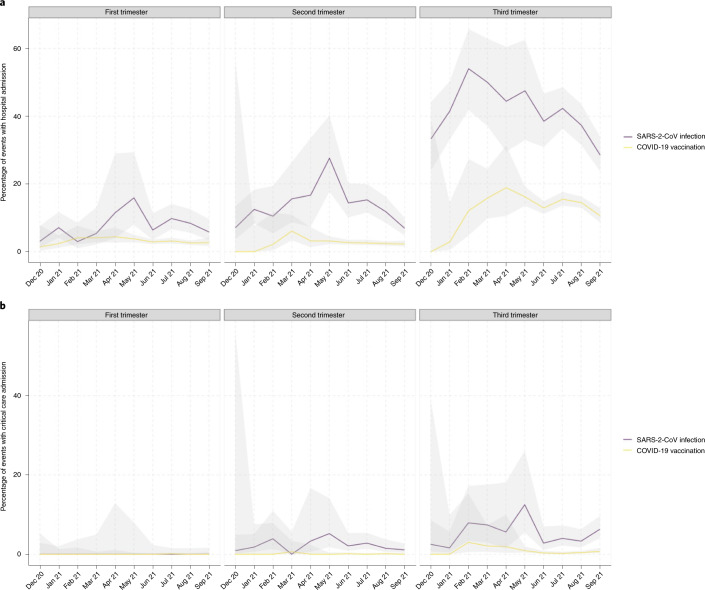
Hospital and critical care admissions after SARS-CoV-2 and COVID-19 vaccination in pregnancy. **a**, Percentage of cases of SARS-CoV-2 infections in pregnancy associated with hospital admission (defined as hospital admission ≤14 d after the date of diagnosis of SARS-2-CoV infection or if the date of diagnosis occurred at any point during a hospital admission) and COVID-19 vaccinations in pregnancy associated with hospital admission (defined as hospital admission ≤14 d after the date of COVID-19 vaccination) occurring 1 December 2020 to 30 September 2021. **b**, Percentage of SARS-CoV-2 infections in pregnancy associated with critical care admission (defined as critical care admission ≤21 d after the date of diagnosis of SARS-CoV-2 infection or if the date of diagnosis occurred at any point during a critical care admission) and COVID-19 vaccinations in pregnancy associated with critical care admission (defined hospital admission ≤21 d after the date of COVID-19 vaccination) occurring 1 December 2020 to 30 September 2021. Definitions are as per Public Health Scotland standards for associated hospital admission and critical care admission. Data are restricted to infections and vaccinations occurring up to 30 September 2021 due to incomplete follow-up times.

A strength of this study is that it used population data with high data completeness. Another strength is that the data describe changes in vaccine uptake over time, with most contemporary data available included. A caveat is that due to source data latency, the most recent 3 months’ pregnancy data may be less complete (there may be some under-ascertainment of the most recent pregnancies and end of pregnancy outcomes). A limitation of our study is that the data presented are descriptive and we have not adjusted for the potential confounding influence of demographics, obstetric or medical conditions. A fully adjusted analysis of all pregnancy outcomes (including early pregnancy outcomes) is planned^27^ when a greater number of end of pregnancy events have accumulated to ensure sufficient power, with appropriate historic comparison groups included^28^. Another limitation of our study is that we were unable to differentiate between hospital and critical care admissions due to SARS-CoV-2 infection from admissions for obstetric care with coincidental SARS-CoV-2 infection. SARS-CoV-2 infection in pregnancy may require admission in its own right for COVID-19 treatment; may trigger obstetric complications that require admission for maternity care or critical care (for example pre-eclampsia or delivery) or may be an incidental finding on admission. Due to this complexity, routine population data coding was not sufficiently detailed to identify coincidental infections. Nevertheless, comparison of hospital and critical care admissions within 28 d of SARS-CoV-2 infection, to those within 28 d of COVID-19 vaccination show different patterns (Fig. 6). Given the established safety profile of vaccinations^18–21^, we would anticipate that post-vaccination admissions are likely to be predominantly for obstetric indications. The higher levels of second and third trimester hospital and critical care admissions following SARS-CoV-2 infections compared to vaccinations suggests that management of COVID-19 or its obstetric sequelae is the most likely reason for excess admissions.

Our findings emphasize the need for continued efforts to increase vaccination uptake in pregnant women, especially in younger and more deprived populations^29^. The percentage of pregnant women vaccinated each month has declined since August 2021, reflecting the roll out of the vaccination program and the likelihood that an increasing proportion of women entering pregnancy are fully vaccinated. However, vaccine coverage, although increasing, remains low, with only a minority of pregnant women fully vaccinated by the time of delivery. Vaccine hesitancy in pregnancy thus requires addressing, especially in light of new recommendations for booster vaccination administration 3 months after the initial vaccination course to help protect against new variants such as Omicron^30^. Low levels of vaccine uptake in Scotland are likely to be representative of those in the United Kingdom and other high-income countries^31^ (Public Health Wales, personal communication). Addressing low vaccine uptake in pregnant women is imperative to protect the health of women and babies.

## Methods

### Ethics and data governance

COPS is a sub-study of EAVE II, using unconsented data that is covered by National Research Ethics Service Committee, South East Scotland 02 approval reference REC 12/SS/0201: SA 2. COPS has been approved by the Public Benefit and Privacy Panel approval reference 2021-0116. Public Health Scotland and the Chief Medical Officer for Scotland are both (independent) data controllers for the national Abortion Act Scotland (AAS) database of termination of pregnancy notifications, thus the Chief Medical Officer has been informed of the use of AAS records. All data were housed within a secure trusted research environment within Public Health Scotland and accessed only by approved researchers.

### Study design and population

COPS is a prospective dynamic pregnancy cohort, identifying all ongoing and completed pregnancies in Scotland, United Kingdom, since 1 March 2020 (ref. ^24^). In this manuscript we used the COPS database as updated in mid-November 2021 linked to records of COVID-19 vaccinations delivered and SARS-CoV-2 infections diagnosed up to and including 31 October 2021. Further detail on the datasets used are in the published protocol^24^. The COPS data dictionary is available online^28^.

### Maternity data sources and linkage

Ongoing pregnancies were identified using antenatal booking appointment data. Completed pregnancies were identified using multiple sources, specifically general practitioner records, general acute hospital discharge records, Scottish Morbidity Record (SMR) 01; maternity hospital discharge records, SMR 02; notification of termination data as governed by AAS; National Records of Scotland (NRS) statutory live birth registrations; NHS Scotland live births; and NRS statutory stillbirth registrations. These datasets capture miscarriage, molar pregnancy or ectopic pregnancy requiring any general practitioner or hospital-based care, a termination of pregnancy, a live birth or a stillbirth.

The CHI number is a unique patient identifier used across all health records in Scotland, enabling integration of healthcare data. All source data incorporated into COPS were subject to a CHI-seeding process in which reported CHIs are reconciled to a contemporaneous unique patient identifier to ensure accurate linkage across sources.

Where multiple records within a data source pertained to the same woman, records were subject to event resolution, in which an iterative grouping procedure assigned records occurring within 83 d (less than 12 weeks) to a single healthcare event with its own universally unique identifier. The estimated date of conception was used to resolve records to a single pregnancy with a single universally unique identifier when gestation information was available and multiple records within a data source pertained to the same woman. Following event resolution, records from each individual data source were linked together to create a comprehensive record for each pregnancy and birth.

The COPS cohort is updated monthly and although source data latency means there is some instability in data for the most recent months, data are generally complete for conceptions and end of pregnancy events occurring up to 3 months previously.

National resources for SARS-CoV-2 infection data and COVID-19 vaccination data were incorporated into the study using CHI linkage.

### Statistical Analysis

Results are based on simple descriptive statistics, for example calculation of the proportion of vaccination events occurring by trimester, or the extended perinatal mortality rate following maternal infection, with relevant definitions provided. Wilson score estimates were used for 95% confidence intervals. All analyses were performed in R version 3.6.1.

Infection and vaccination in pregnancy was defined as infection diagnosed or vaccination given, at any point from the date of conception (2 + 0 weeks gestation) to the date the pregnancy ends inclusive (censoring infections and vaccinations occurring at 44 + 0 weeks gestation or over as it is very likely that these women have completed their pregnancy, but the end of pregnancy record has not yet been received by Public Health Scotland). The date of first positive viral RT−PCR sample collection was taken as the date of onset of the first episode of COVID-19. Subsequent episodes were recorded if a positive viral RT−PCR sample was taken ≥90 d after a first positive sample. Lateral flow test results were not considered. For the duration of our study period, anyone in Scotland having a positive lateral flow test was advised to have a follow up RT-PCR test to confirm COVID-19.

Vaccination status was defined as unvaccinated (no previous COVID-19 vaccination before the date of onset of COVID-19 or with one dose of vaccination ≤21 d before the date of onset), partially vaccinated (one dose of vaccination >21 d before the date of onset of COVID-19 or two doses of vaccination with the second dose ≤14 d before the date of onset) or fully vaccinated (two doses of vaccination with the second dose >14 d before the date of onset of COVID-19).

Monthly vaccine uptake was calculated as the number of pregnant women vaccinated during the month divided by the number of women with ongoing pregnancies at the start of the month of interest. Coverage of vaccination at delivery, was calculated as the proportion of women who, at the time of giving birth, have been vaccinated, whether vaccination was before or during pregnancy. Uptake in the general female population of reproductive age (18−44 years) was calculated as the number of women 18−44 years of age vaccinated during the month divided by the total number of women 18−44 years of age. Coverage in the general female population of reproductive age (18−44 years) was calculated as the cumulative number of women 18−44 years of age vaccinated by the end of each month divided by the total number of women 18−44 years of age. Numbers of women 18−44 years were derived from 2020 mid-year population estimates^26^. Although pregnant women 11−55 years of age were included in the COPS cohort, the comparator group was restricted to women 18−44 years. This is the Public Health Scotland standard definition of women of reproductive age and helps avoid bias in comparisons that could result from very low numbers of pregnancies at the extremes of reproductive age.

National hospital discharge records were linked to pregnancy SARS-CoV-2 RT−PCR testing data to identify women who were admitted to hospital around the time of infection. Admissions to general acute units (SMR 01) and maternity units (SMR 02) were included. SARS-CoV-2 infection was defined as associated with a hospital admission if the woman was admitted to hospital ≤14 d after the date of onset of COVID-19 or if the date of onset occurred at any point during a hospital admission (as per Public Health Scotland standard definition). National critical care discharge records from the Scottish Intensive Care Society Audit Group were used to identify women who were admitted to critical care. Completed admissions to all intensive care units and general (non-obstetric) high-dependency units across Scotland were included. Completed admissions to the seven obstetric high-dependency units that contribute data to Scottish Intensive Care Society Audit Group (collectively covering around 60% of deliveries in Scotland) were also included. SARS-CoV-2 infection was defined as associated with a critical care admission if the woman was admitted to critical care ≤21 d after the date of onset of COVID-19 or if the date of onset occurred at any point during a critical care admission (as per Public Health Scotland standard definition).

### Comparator data

Comparator data from the general population of women of reproductive age (taken as 18−44 years for these analyses) were obtained from Public Health Scotland Open data with mid-year population estimate denominators (2020)^26^.

### Data quality and completeness

The national Scottish data used within this study undergo regular assessment and quality assurance with high level of completeness and accuracy^32,33^. The CHI number was used to link the SARS-CoV-2 PCR testing data and COVID-19 vaccination records to the pregnancy records. Quality of linkage was assured by the testing, vaccination and pregnancy records including complete and accurate information on individuals’ CHI number, as confirmed in the Results section.

### Reporting Summary

Further information on research design is available in the Nature Research Reporting Summary linked to this article.

## Online content

Any methods, additional references, Nature Research reporting summaries, source data, extended data, supplementary information, acknowledgements, peer review information; details of author contributions and competing interests; and statements of data and code availability are available at 10.1038/s41591-021-01666-2.

## Supplementary information

Reporting Summary

## Data Availability

Aggregate data files of infections among pregnant women and the general population and vaccinations delivered to pregnant women and the general population are available here: https://www.opendata.nhs.scot/organization/health_protection. Patient-level data underlying this article cannot be shared publicly due to data protection and confidentiality requirements. Public Health Scotland and the Chief Medical Officer for Scotland are the data holders for the data used in this study. Data can be made available to approved researchers for analysis after securing relevant permissions from the data holders via the Public Benefit and Privacy Panel. Enquiries regarding data availability should be directed to phs.edris@phs.scot.

## Code availability

Metadata and code are available at https://github.com/Public-Health-Scotland/COPS-public.
