# Supplementary material for: SARS-CoV-2 infection and COVID-19 vaccination rates in pregnant women in Scotland
Source: Nat Med. 2022 Jan 13;28(3):504–12. doi: 10.1038/s41591-021-01666-2 (PMC8938271; doi:10.1038/s41591-021-01666-2)
Supplement: Supplementary file 1 — Reporting Summary [file 41591_2021_1666_MOESM1_ESM.pdf]

nature portfolio

Corresponding author(s): Sarah J Stock

Last updated by author(s): Dec 20, 2021

Reporting Summary

Nature Portfolio wishes to improve the reproducibility of the work that we publish. This form provides structure for consistency and transparency in reporting. For further information on Nature Portfolio policies, see our Editorial Policies and the Editorial Policy Checklist.

Statistics

For all statistical analyses, confirm that the following items are present in the figure legend, table legend, main text, or Methods section.

n/a	Confirmed	
☐	☒	The exact sample size (*n*) for each experimental group/condition, given as a discrete number and unit of measurement
☒	☐	A statement on whether measurements were taken from distinct samples or whether the same sample was measured repeatedly
☒	☐	The statistical test(s) used AND whether they are one- or two-sided<br>*Only common tests should be described solely by name; describe more complex techniques in the Methods section.*
☒	☐	A description of all covariates tested
☒	☐	A description of any assumptions or corrections, such as tests of normality and adjustment for multiple comparisons
☒	☐	A full description of the statistical parameters including central tendency (e.g. means) or other basic estimates (e.g. regression coefficient) AND variation (e.g. standard deviation) or associated estimates of uncertainty (e.g. confidence intervals)
☒	☐	For null hypothesis testing, the test statistic (e.g. *F*, *t*, *r*) with confidence intervals, effect sizes, degrees of freedom and *P* value noted<br>*Give P values as exact values whenever suitable.*
☒	☐	For Bayesian analysis, information on the choice of priors and Markov chain Monte Carlo settings
☒	☐	For hierarchical and complex designs, identification of the appropriate level for tests and full reporting of outcomes
☒	☐	Estimates of effect sizes (e.g. Cohen's *d*, Pearson's *r*), indicating how they were calculated

Our web collection on statistics for biologists contains articles on many of the points above.

Software and code

Policy information about availability of computer code

Data collection	R version 3.6.1
Data analysis	R version 3.6.1

For manuscripts utilizing custom algorithms or software that are central to the research but not yet described in published literature, software must be made available to editors and reviewers. We strongly encourage code deposition in a community repository (e.g. GitHub). See the Nature Portfolio guidelines for submitting code & software for further information.

Data

Policy information about availability of data

All manuscripts must include a data availability statement. This statement should provide the following information, where applicable:

- Accession codes, unique identifiers, or web links for publicly available datasets
- A description of any restrictions on data availability
- For clinical datasets or third party data, please ensure that the statement adheres to our policy

Data files of infections among pregnant women and the general population and vaccinations delivered to pregnant women and the general population are available here: https://www.opendata.nhs.scot/organization/health_protection.
Other personal data underlying this article cannot be shared publicly as they are sensitive. Public Health Scotland and the Chief Medical Officer for Scotland are the data holders for the data used in this study. Data are available to researchers for analysis after securing relevant permissions from the data holders. Enquiries regarding data availability should be directed to phs.edris@phs.scot.

Field-specific reporting

Please select the one below that is the best fit for your research. If you are not sure, read the appropriate sections before making your selection.

☒ Life sciences ☐ Behavioural & social sciences ☐ Ecological, evolutionary & environmental sciences

For a reference copy of the document with all sections, see nature.com/documents/nr-reporting-summary-flat.pdf

Life sciences study design

All studies must disclose on these points even when the disclosure is negative.

Sample size	This is a descriptive study using routinely collected health data. No sample size calculation was performed.
Data exclusions	Data were excluded if there were pre-specified unfeasible values, as described in the data dictionary available from https://github.com/Public-Health-Scotland/COPS-public.
Replication	N/A - descriptive study of routinely collected health data.
Randomization	N/A - descriptive study of routinely collected health data.
Blinding	N/A - descriptive study of routinely collected health data.

Reporting for specific materials, systems and methods

We require information from authors about some types of materials, experimental systems and methods used in many studies. Here, indicate whether each material, system or method listed is relevant to your study. If you are not sure if a list item applies to your research, read the appropriate section before selecting a response.

Materials & experimental systems

n/a	Involved in the study	
☒	☐	Antibodies
☒	☐	Eukaryotic cell lines
☒	☐	Palaeontology and archaeology
☒	☐	Animals and other organisms
☐	☒	Human research participants
☒	☐	Clinical data
☒	☐	Dual use research of concern

Methods

n/a	Involved in the study	
☒	☐	ChIP-seq
☒	☐	Flow cytometry
☒	☐	MRI-based neuroimaging

Human research participants

Policy information about studies involving human research participants

Population characteristics	Women (15 to 55 years) who had early pregnancy loss, termination of pregnancy, live or stillbirth, neonatal death, or antenatal care for an ongoing pregnancy.
Recruitment	National, prospective dynamic cohort of routinely collected healthcare data.
Ethics oversight	COPS is a sub-study of EAVE II, using unconsented data, which is covered by National Research Ethics Service Committee, South East Scotland 02 approval reference REC 12/SS/0201: SA 2. COPS has been approved by the Public Benefit and Privacy Panel approval reference 2021-0116. Public Health Scotland and the Chief Medical Officer for Scotland are both (independent) data controllers for the national Abortion Act Scotland (AAS) database of termination of pregnancy notifications, thus the Chief Medical Officer has been informed of the use of AAS records for this study.

Note that full information on the approval of the study protocol must also be provided in the manuscript.
